# Speed-Accuracy Dissociation in Fencing: Performance Fatigability Without a Detectable Decline in Touch Accuracy: An Exploratory Study

**DOI:** 10.3390/sports14100459

**Published:** 2026-10-15

**Authors:** Mirko Colombo, Gian Mario Migliaccio, Domenico Martone, Johnny Padulo

**Affiliations:** 1 Department of Biomedical Sciences for Health (SCIBIS), Università degli Studi di Milano, 20133 Milan, Italy; mirkofit04@gmail.com (M.C.); johnny.padulo@unimi.it (J.P.); 2 Department of Human Sciences and Promotion of the Quality of Life, San Raffaele Rome Open University, 00166 Rome, Italy; gianmario.migliaccio@uniroma5.it; 3 Department of Economics, Law, Cybersecurity and Sports Sciences, University Parthenope, 80035 Naples, Italy

**Keywords:** fencing, repeated sprint ability, performance fatigability, accuracy, precision, heart rate, countermovement jump, test shortening, preliminary validation

## Abstract

Background: It remains unclear whether physiological load affects fencing accuracy to the same extent as movement speed and whether a shortened protocol can reproduce the outcomes of a full repeated-sprint protocol. Methods: Ten fencers (foil/épée; 7 men, 3 women; 16–30 years) completed anthropometric, handgrip strength, and maximal heart rate assessments on Day 1; a target-hitting task at baseline and at 50% and 85% of maximal heart rate using two fencing gestures on Day 2; and ten 16 m shuttles (4–2–4–2–4 m), separated by 8 s of recovery and each ending with a lunge to a graded target, on Day 3. Results: Shuttle time increased non-linearly, reaching an estimated maximum at shuttle 7.27 (95% CI: 6.15–11.34), whereas no significant trend in touch performance was detected across shuttles (*p* = 0.988). On Day 2, accuracy did not differ across heart rate conditions but differed between gestures (*p* < 0.001). On Day 3, heart rate reached 84.60–93.45% of maximal heart rate. The seven-shuttle fatigue index showed close agreement with the full-protocol index (ICC = 0.96; limits of agreement: −2.58 to 1.25), indicating reproducibility rather than criterion validity. Conclusions: Performance fatigability was expressed as slower movement, with no detectable decline in touch accuracy; the underlying physiological or neuromuscular mechanisms were not assessed. Seven shuttles emerged as a candidate protocol length, which requires test-retest reliability and independent validation before it can replace the full protocol.

## 1. Introduction

The number of repetitions used in a repeated-sprint ability (RSA) protocol is rarely chosen on the basis of direct evidence that it is necessary; it is more often inherited from earlier protocols in a related sport. Changing the structural parameters of an RSA test, not only its overall duration, changes what the test measures: altering the number of directional changes per sprint alters both mechanical output and metabolic demand [1], and comparing one against two, or single- against multi-directional, changes of direction [2], or altering the curvature [3] or the angle [4,5] of the sprint path, yields protocols that are related but not interchangeable.

Fencing adds a dimension that pure locomotor RSA protocols do not capture: each shuttle in a fencing-specific test ends not in a line to cross but in a lunge, a graded motor skill whose outcome carries the competitive value of the action [6,7]. Whether accumulating fatigue degrades this technical outcome to the same extent that it degrades speed is not trivial. In this context, fatigue is operationalised as performance fatigability, that is, the decline in an objective measure of performance over a discrete period of activity, which by itself does not identify the physiological or neuromuscular mechanisms responsible for the decline [8]. Using an instrumented lunge simulator, fencers facing increasing task difficulty were found to become significantly slower while largely preserving touch accuracy, consistent with a speed-accuracy trade-off in which the athlete protects the technical outcome at the expense of speed [9,10,11]. This protective capacity may be level-dependent: precision loss under physical fatigue has been reported as significant in sub-elite but not Olympic-level fencers performing the same protocol [12], and elite fencers respond significantly faster than novices on a standardised light-based target task across attack types [13], and stimulus uncertainty itself modulates the reaction response of fencers [14]. Perceptual-cognitive qualities are also trainable in young fencers, although responses vary markedly between individuals, as recently shown for choice reaction time in U20 foil fencers [15]. Not every study agrees: a significant accuracy decline after a fatiguing protocol has also been reported in a small sample of city-level épée fencers [16].

Exertion does not degrade the accuracy of technical actions uniformly: tennis groundstroke accuracy was equivalent to that at rest under moderate fatigue but declined markedly after high-intensity fatigue, in both expert and non-expert players [17]. A separate literature on basketball shooting accuracy across graded, staged heart-rate conditions offers a specific, testable pattern: accuracy is preserved from rest to a moderate relative heart rate but drops sharply beyond a higher threshold, consistently for the three-point shot [18] and the jump-shot [19] (both *p* < 0.01 at 80–85%HRmax versus rest, both non-significant at 50%HRmax). Whether an analogous threshold applies to a fencing-specific technical action under directly comparable staged conditions has not, to our knowledge, been tested.

If fatigue-index metrics are to be trusted, their known measurement instability must also be taken seriously: the formula in common use [20] expresses fatigue as a small relative difference between total and best time, a construction that is mathematically predisposed to instability [21] and has produced coefficients of variation as high as 50% across published RSA studies [22], and a systematic review has confirmed the weak absolute and relative reliability of the fatigue index compared with total and best sprint time [23]. This motivates asking whether a shorter protocol reproduces the same signal with fewer repetitions, particularly since the number of sprints in published RSA protocols ranges from 3 to 15 with little standardisation [24]. Lower-limb power is an established correlate of repeated-sprint fatigue resistance [25,26], and a fencing-specific change-of-direction test built on precisely the 4/2/4/2/4 m shuttle geometry used in the present protocol correlates moderately to very largely with countermovement jump, squat jump, sprint time, and isokinetic strength, and discriminates high-ranked from low-ranked fencers [27].

None of the constructs this study addresses, a joint speed-accuracy-precision profile of fatigue, a shortened fatigue index proposed as a candidate abbreviation of the full protocol, and the shuttle-with-target test itself, can be validated against a single external criterion, since none exists for a fencing-specific composite of this kind. The present approach is therefore grounded in construct validity: the demonstration that each measure behaves in a manner theoretically consistent with the construct it represents [28]. This rationale also motivates the breadth of variables measured. “Fencing-specific repeated-sprint capacity” is not a single quantity: it is expressed jointly through movement speed, technical accuracy, technical precision, the physiological cost at which these are produced, and the underlying neuromuscular power that supports repeated efforts. A single outcome variable cannot distinguish an athlete whose fatigue-related decline is speed-limited from one whose decline is precision-limited or power-limited, a distinction with different implications for training prescription. Measuring time, score-derived accuracy and precision, heart rate, and jump-derived lower-limb power together, and testing whether they converge in theoretically expected directions, is itself the strategy pursued here, tested through three complementary sources of evidence: agreement between a shortened and the full-protocol fatigue index, a nested-measure comparison rather than validation against an independent criterion; exploratory correlation of the shortened index with independently measured lower-limb power (convergent validity); and correlation between target performance measured on the continuous, fatiguing Day 3 shuttle protocol and target performance measured on the controlled, staged Day 2 task (convergent validity across two independent assessments of the same technical skill).

This study therefore describes, across a three-day protocol, the joint trajectory of shuttle time and touch score under both a controlled, staged physiological load (Day 2) and a continuous fencing-specific repeated-shuttle task (Day 3), identifies by simulation a candidate minimum number of shuttles that reproduces the Day 3 fatigue signature and its association with lower-limb power, and reports the convergent-validity evidence available for the shuttle-with-target test from the variables collected across all three days. Given the sample size, all athletes were analysed as a single group throughout, rather than split by competitive category, weapon, or sex.

## 2. Materials and Methods

### 2.1. Participants

Ten competitive fencers (seven men, three women; age range 16 to 30 years; Table 1) took part in the study, recruited from two fencing clubs between March and June 2026. Athletes competed in foil or épée, spanning the Cadetti, Giovani, Under-23, and Assoluti national age categories, with national ranking positions ranging approximately from 5th to 300th and competitive experience of approximately 5 to 6 years; seven athletes were right-handed and three left-handed.

This study was approved by the local Ethics Committee of the Università degli Studi di Milano (no. 2/26, 20 January 2026) and was performed in accordance with the ethical standards [29]. All participants provided written informed consent in accordance with the Declaration of Helsinki and were free to withdraw from the study at any time; for participants who were minors, written informed consent was provided by a parent or legal guardian.

### 2.2. Overall Design

Testing was completed over three separate days. Day 1 comprised anthropometric measurement, a handgrip strength test, and determination of maximal heart rate and estimated V̇O_2_max. Day 2 comprised a randomised precision and accuracy task at the target, performed at three physiological conditions and two gesture types. Day 3 comprised the fencing-specific repeated-shuttle test. Before each testing session, athletes completed a standardised 10-min warm-up comprising joint mobility exercises, coordinative running, jumps, sprints, and fencing-specific technical gestures. All trials on Days 2 and 3 were video recorded and analysed as described below.

Body mass was recorded with a digital electronic scale (precision 0.1 kg) and height with a graduated tape measure. Leg length was determined as the distance between the greater trochanter and the medial malleolus. Handgrip strength was assessed in a standing position with a digital dynamometer (InGrip, InBody Co., Ltd., Seoul, Republic of Korea), two trials per hand with 3 min of passive recovery between trials; the highest value per hand was retained. Maximal heart rate was determined using the 20 m Shuttle Run Test (Beep Test [30]), administered and scored with the Beep Test application (Effortless Code Limited, London, UK), with the 20 m distance measured using a Zozen measuring wheel (Zozen Tools Co., Ltd., Zhejiang, China) and heart rate monitored continuously with a Polar H10 (Polar Electro Oy, Kempele, Finland [31]) or Garmin HRM 600 (Garmin Ltd., Olathe, KS, USA) chest strap; the highest value recorded during the test was taken as maximal heart rate, which defined the 50% and 85% heart-rate conditions used on Day 2. This field-based, single-session value is an operational estimate rather than a laboratory-, gas-exchange-verified maximum, a distinction returned to in the Discussion. Estimated V̇O_2_max was derived from Beep Test performance using the same application [30,32].

### 2.3. Precision and Accuracy Target Task (Day 2) and Target Apparatus

Precision and accuracy were assessed with a target constructed specifically for this study, consisting of five concentric circles of textile material, hand-sewn, with diameters of 5, 10, 15, 20, and 25 cm, scored 10, 8, 6, 4, and 2 points from centre to periphery; hits outside the target area scored 0. The target was sewn inside a shirt worn by a mannequin or a human partner (Figure 1A,B).

Execution distance was not standardised but adapted to each athlete’s individual characteristics and to the technical gesture required, to reproduce conditions as close as possible to fencing practice; this is a disclosed methodological limitation, addressed further in the Discussion.

On Day 2, athletes performed the target task under three exercise-intensity states, operationally defined by the heart rate recorded at their onset: baseline (recorded before warm-up, at rest), 50%HRmax (recorded immediately after the standardised warm-up), and 85%HRmax (reached via sport-specific shuttles until the target heart rate was attained, at which point trials began immediately), each monitored continuously with the same heart-rate monitors used on Day 1; heart rate during the five trials themselves was not independently re-verified against the target condition and may have drifted from the value recorded at onset. Because the 50%HRmax and 85%HRmax states were also reached through different preceding exercise exposures (standardised warm-up versus sport-specific shuttles), they are interpreted as approximate exercise-intensity states rather than precisely controlled physiological conditions. The staged design follows the paradigm used to test basketball shooting accuracy at rest and at approximately 50% and 80–85% of maximal heart rate [18,19], so that the Day 2 results can be compared directly with that literature. Two gesture types were assessed at each condition, hereafter standardized as the “straight thrust” and the “lunge”: the straight thrust is a direct hit delivered from the en-garde position by simple extension of the armed limb, without trunk or lower-limb displacement; the lunge is a hit delivered with rapid extension of the armed limb combined with forward projection of the body through extension of the rear leg and advancement of the front leg. Five trials were planned for each condition-by-gesture combination; the realized trial count varied slightly (43 of 60 condition-by-gesture cells had exactly five trials, 14 had six, and one had seven, and one athlete’s baseline condition was not recorded for either gesture), giving 306 trials in total rather than the nominal 300. These deviations from the planned protocol arose during field data collection: additional executions within a block were recorded rather than discarded, and the baseline block of one athlete was not recorded. No trial was excluded post hoc, and the unequal number of trials per cell was accommodated by the mixed-effects models, which use all available trials with a random intercept per athlete.

Each athlete completed ten consecutive shuttles over a 16 m course divided into five segments (4–2–4–2–4 m; Figure 1C), starting from a static position on verbal signal from the examiner; this shuttle geometry follows the protocol described by Chtara et al. [27] for fencing-specific change-of-direction assessment. At the completion of every shuttle, athletes performed a lunge to the same graded target described above, scored on the same 0–10 scale. Passive recovery between shuttles was fixed at 8 s. This value is shorter than the average stoppage duration reported between exchanges in elite competitive bouts across weapons (15.1 to 16.0 s [33]) and was adopted deliberately as an abbreviated interval, consistent with standard RSA testing practice of imposing shorter-than-competitive recovery to elicit a measurable fatigue response within a limited number of repetitions [34,35]. With shuttles lasting approximately 9 s, the resulting work-to-recovery ratio (approximately 1:0.9) matches that reported for elite épée (mean work duration 17.9 s; W:R 1:0.9) more closely than that of foil (5.8 s; W:R 1:2.6) [33]; the test is therefore fencing-specific in its movement structure, but it does not reproduce the competitive work-recovery demands of both weapons. Heart rate was monitored continuously throughout, and mean and peak values were recorded. Rating of perceived exertion (Borg CR10 scale [36]) was recorded digitally immediately before the session and again 30 min after its completion.

### 2.4. Video Recording and Kinematic Analysis

All Day 2 and Day 3 trials were video recorded by a dedicated operator using an iPhone 15 (Apple Inc., Cupertino, CA, USA) in slow-motion mode at 120 Hz, mounted on an OBO T-255 tripod (OBO, Zhongshan, Guangdong, China) at maximal height to allow simultaneous visualisation of the athlete and the target. Recordings were analysed frame-by-frame with Kinovea (version 2025.2; Kinovea Open-Source Project, Bordeaux, France [37]). For upper-limb parameters, movement onset was identified as the first frame showing observable wrist movement; action end was identified as the frame corresponding to contact between the weapon tip and the target. For lower-limb coordination, the temporal difference between first observable wrist movement and first observable foot movement was additionally computed. From this analysis, reaction time, total execution time, autonomous execution time, the upper–lower limb temporal difference, and mean execution velocity were derived.

### 2.5. Outcome Measures

Two technical outcomes were analysed: mean touch score (0–10 points), which is the index of accuracy, and precision, defined throughout as the within-athlete sample standard deviation of scores across the trials contributing to a given estimate (across the five trials within each Day 2 condition-by-gesture cell, or across all ten Day 3 shuttles for a given athlete). Accuracy was also expressed as the mean absolute error from the maximum score (MAE = Σ|score − 10|/n, points); because no score can exceed 10, MAE is equal to 10 minus the mean touch score. MAE is therefore a linear rescaling of the mean score, expressed as distance from the maximum, and is not interpreted as an indicator of performance separate from it. This within-athlete precision is conceptually distinct from between-athlete dispersion, that is, how much athletes differ from one another at a given shuttle or condition, which is reported separately in Results where relevant and is not itself termed “precision” to avoid conflating the two. Because the 0 to 10 outcome is a discrete, zone-based score rather than a continuous measure of spatial error, the mean score (or MAE) and the SD are score-based proxies for accuracy and precision, respectively, and not direct measures of radial error or spatial dispersion; with approximately five trials per Day 2 cell, the SD-based estimate of precision is particularly imprecise. A composite Performance Error Index (PEI) was computed as PEI = √(MAE^2^ + SD^2^), that is, √((10 − mean score)^2^ + SD^2^), by analogy with the total error index used in target-aiming tasks, which combines the systematic and variable components of error into a single value [38], with lower values indicating better performance quality; because PEI for the Day 2 cells is derived from only five trials, it is treated as an exploratory composite summarising, in one value, an off-centre and an inconsistent touch, rather than as a validated metric. For Day 3 specifically, time for each shuttle segment and their sum (T_TOT), mean shuttle velocity, and mean and peak heart rate were also recorded for every shuttle. The fatigue index (FI) was computed following Fitzsimons et al. [20]: FI = 100 × (ΣT_TOT/(best time × number of shuttles) − 100. This formula is known to be statistically unstable [21,22,23], and it was not treated here as a validated absolute measure of fatigue; rather, it was used as a pre-specified, deterministic ranking criterion to test whether a shortened protocol reproduces the athlete ordering generated by the conventional full-protocol calculation, a narrower and more defensible claim than validating FI itself.

### 2.6. Jump and Neuromuscular Assessment

In a session distinct from the three testing days, each athlete performed a squat jump (SJ), a bilateral countermovement jump (CMJ), and unilateral countermovement jumps on the dominant and non-dominant leg, assessed with the My Jump Lab application (Carlos Balsalobre-Fernández, Madrid, Spain [39]). Athletes kept their hands on their hips throughout to minimise upper-limb contribution. For SJ, athletes held a semi-squatted position for approximately 3 to 4 s before jumping, to minimise the contribution of the stretch-shortening cycle; for CMJ, athletes performed a rapid countermovement immediately followed by a maximal vertical push-off [40]. Two trials were performed per test with 3 min of passive recovery, and the best value was retained. From these tests, a between-limb asymmetry index (My Jump Lab, from the unilateral CMJ difference between dominant and non-dominant leg [41,42]), an Elasticity Index, conceptually equivalent to the eccentric utilisation ratio (Elasticity Index (%) = ((CMJ − SJ)/CMJ) × 100 [43]), and the CMJ/SJ ratio (CMJ height/SJ height [43]) were derived. Musculotendinous stiffness (%) was also recorded. Values of exactly 0, physiologically implausible for the variables concerned, were treated as not measured rather than as true observations.

### 2.7. Statistical Analysis

All analyses were performed in Python (version 3.12; Python Software Foundation, Wilmington, DE, USA [44]), using SciPy (version 1.11 [45]) and statsmodels (version 0.14 [46]) for statistical modelling. For Day 3, trends across shuttles for time and score were modelled with linear mixed-effects models, maximum likelihood estimation, shuttle number (1–10) as a continuous fixed effect and a random intercept per athlete; a quadratic term was added to the time model and compared against the linear model with a likelihood-ratio test, and the 95% confidence interval of the quadratic vertex was obtained by non-parametric bootstrap (500 athlete-level resamples with replacement, re-fitting the quadratic model in each resample). For Day 2, trial-level absolute error from the maximum score (10 − score) was modelled with a linear mixed-effects model with heart-rate condition (categorical, reference = baseline), gesture type, and their interaction as fixed effects and a random intercept per athlete; the interaction term was tested with a likelihood-ratio test against the corresponding additive model. Because absolute error is 10 minus the score, the same model fitted to touch score gives identical *p*-values with coefficients of opposite sign, so positive coefficients indicate a lower score. Associations between accuracy or precision and mean or peak heart rate were assessed with Spearman’s rho. A Benjamini-Hochberg correction was applied across the primary family of Day 3 trend tests (shuttle time, touch score, and between-athlete dispersion; absolute error was not tested separately, because it mirrors touch score), and separately across the family of convergent-validity correlations described below.

For the shuttle-number simulation, the fatigue index was recomputed for each athlete using only the first k shuttles, for every k = 2 to 10. Because the shortened index is nested within the full-protocol index (the first k shuttle times are a subset of the ten), their Spearman correlation is reported as agreement, not concurrent validity. Agreement between the k = 7 index, the length ultimately proposed, and the full-protocol index was characterised in more detail with the intraclass correlation coefficient (ICC, two-way random effects, absolute agreement, single measures [47]) and the Bland-Altman method (mean bias and 95% limits of agreement). Associations between the shortened index and each jump or physiological variable were tested at two levels: a single, pre-specified test per variable at the current protocol length (k = 10), corrected for the resulting family of comparisons with Benjamini-Hochberg; and an exploratory, post hoc search across all nine values of k for each variable, with the resulting nine *p*-values corrected for multiplicity within that variable’s own search. This search is reported as hypothesis-generating.

Convergent validity of the Day 3 shuttle-with-target test was assessed by correlating athlete-level Day 3 accuracy, precision, and PEI with the corresponding athlete-level Day 2 values (pooled across heart-rate conditions and gesture types), and by correlating Day 3 PEI with Day 1 physical capacity measures (dominant- and non-dominant-hand grip strength, estimated V̇O_2_max). These six correlations were treated as a single pre-specified family and corrected with Benjamini-Hochberg. A sensitivity analysis excluded the single athlete with a constant zero score across all ten Day 3 shuttles.

Given the sample size (*n* = 10 athletes), a within-subject, paired-comparison design, an alpha of 0.05 (two-sided), and a target power of 0.80, approximately 34 athletes would be required to detect a medium effect (Cohen’s dz = 0.50 [48]), 15 to detect a large effect (dz = 0.80), and 10 to detect a very large effect (dz = 1.00). With the present sample of 10, achieved power was 0.62 for a large effect and 0.80 for a very large effect, and the minimum detectable effect size at 80% power was dz = 1.00; this is also why competitive category, weapon, and sex were not used to split the sample. The study is therefore described throughout as exploratory, capable of detecting only very large within-subject effects [49,50], and non-significant results are not interpreted as evidence of a true null effect. Because no equivalence margin (smallest effect size of interest) was specified a priori, a non-significant trend is reported as the absence of a detectable change and not as evidence that performance was maintained within a practically acceptable range [51]. Statistical significance was set at *p* < 0.05 throughout, and exact *p*-values are reported to three decimal places except where *p* < 0.001; all descriptive statistics are reported to a minimum of two decimal places.

## 3. Results

### 3.1. Precision and Accuracy Under Graded Physiological Load

Touch accuracy did not differ significantly between heart-rate conditions (trial-level mixed model on absolute error from the maximum score; 50%HRmax vs. baseline: β = 0.250, *p* = 0.219; 85%HRmax vs. baseline: β = 0.032, *p* = 0.877), despite adequate trial-level sample size (*n* = 306 trials; see Methods). Gesture type had a significant effect: the straight thrust showed significantly lower deviation from the maximum score than the lunge (β = −0.579, *p* < 0.001; Table 2), and the two gestures also differed descriptively in execution time, distance, and velocity (Table 3).

The upper–lower limb temporal difference was, by definition, uniformly absent for the straight thrust, which involves no lower-limb movement. The heart-rate condition × gesture interaction was not significant (likelihood-ratio χ^2^(2) = 0.91, *p* = 0.634), providing no evidence that the two gestures responded differently to heart-rate condition.

### 3.2. Speed and Touch Score Across the Repeated-Shuttle Protocol

#### Descriptive Trajectory and Time Course

Mean shuttle time rose from 8.91 ± 0.56 s at shuttle 1 to 9.59 ± 1.08 s at shuttle 7 (shuttle-level statistics in Supplementary Table S1). The full-protocol fatigue index was 7.94 ± 4.13%, mean and peak heart rate were 165.60 ± 7.32 and 182.90 ± 11.42 beats·min^−1^, and RPE (Borg CR10) rose from 0.00 ± 0.00 before the session to 6.10 ± 0.99 at 30 min after it (Supplementary Table S2).

Shuttle time increased significantly across the protocol (β = 0.054 s per shuttle, SE = 0.014, z = 3.78, *p* < 0.001), and a quadratic model fit significantly better than the linear model (quadratic term β = −0.015, *p* = 0.005; likelihood-ratio χ^2^(1) = 7.58, *p* = 0.006). The fitted quadratic trajectory reached its estimated maximum at shuttle 7.27 (bootstrap 95% CI 6.15–11.34; Figure 2).

The width of this interval, which extends beyond the tenth shuttle at its upper bound, indicates that the precise location of the maximum is estimated with limited precision in this sample and should not be read as a sharply defined physiological landmark. A paired comparison of shuttle 10 versus shuttle 1 confirmed the overall effect (+0.53 s, t(9) = 3.44, *p* = 0.007, dz = 1.09), as did a block comparison of the first five versus the last five shuttles (9.20 s vs. 9.48 s, t(9) = 2.45, *p* = 0.037, dz = 0.77).

### 3.3. Touch Score Trajectory and Between-Athlete Dispersion

No significant linear trend across shuttles was detected for touch score (β = 0.001, *p* = 0.988; Figure 3; MAE, being 10 minus the score, necessarily gives the same result); individual athlete trajectories, shown alongside the group mean in Figure 3, varied considerably from shuttle to shuttle, and this absence of a detectable group-level trend should be read against that background heterogeneity rather than as evidence of stable individual performance.

The between-athlete dispersion of score at each shuttle, a distinct quantity from the within-athlete precision defined in Methods, also showed no significant trend across shuttles (*p* = 0.362). Neither the score nor the between-athlete-dispersion trend test survived Benjamini-Hochberg correction (score, q = 0.988; dispersion, q = 0.543), while the time trend remained significant after the same correction (q < 0.001).

### 3.4. Physiological Load

Mean heart rate during the Day 3 test showed a positive association with absolute error from the maximum score (MAE) that did not reach significance (ρ = 0.60, *p* = 0.065); peak heart rate showed a weaker, non-significant association (ρ = 0.28, *p* = 0.428). Expressed relative to each athlete’s own Day 1 maximal heart rate rather than in absolute beats·min^−1^, mean heart rate during Day 3 corresponded to 84.60 ± 3.56% of maximal heart rate and peak heart rate to 93.45 ± 6.00% (Figure 4).

Nine of the ten athletes reached at least 85% of maximal heart rate at some point during the test, and two exceeded their own Day 1 recorded maximum at peak, consistent with a field-based incremental test not always eliciting a true physiological ceiling. The association between this individually normalised %HRmax and accuracy error was materially unchanged from the absolute-heart-rate analysis (mean %HRmax: ρ = 0.58, *p* = 0.077; peak %HRmax: ρ = 0.23, *p* = 0.528).

### 3.5. Convergent Validity of the Shuttle-with-Target Test

Athlete-level Day 3 target performance correlated positively with Day 2 target performance for all three indices, though none reached significance at this sample size (accuracy: ρ = 0.47, *p* = 0.167; precision: ρ = 0.30, *p* = 0.393; PEI: ρ = 0.39, *p* = 0.267; Supplementary Table S3).

Excluding the single athlete with a constant zero score on Day 3, these correlations strengthened but remained non-significant (accuracy: ρ = 0.55, *p* = 0.129; precision: ρ = 0.43, *p* = 0.252; PEI: ρ = 0.44, *p* = 0.232). Day 3 PEI correlated significantly with dominant-hand grip strength before correction (ρ = 0.68, *p* = 0.030), in the direction of higher grip strength corresponding to worse target performance, but this association weakened and lost significance both when the same outlier was excluded (ρ = 0.61, *p* = 0.079) and after Benjamini-Hochberg correction across the six-test convergent-validity family (q = 0.177). No other Day 1 physical measure was associated with Day 3 PEI (non-dominant grip: ρ = −0.11, *p* = 0.763; estimated V̇O_2_max: ρ = −0.28, *p* = 0.463, *n* = 9). None of the six correlations in this family survived correction (minimum q = 0.177); given the achieved power described in Statistical Analysis, this pattern of positive but non-significant associations is not evidence against a real relationship.

### 3.6. Minimum Shuttle Number and Its Relationship to Lower-Limb Power

#### Agreement of the Shortened Fatigue Index with the Full Protocol

Because the shortened index at each k is mathematically nested within the full ten-shuttle index, its Spearman correlation with the full index necessarily increases as k approaches 10 and reaches unity at k = 10; this correlation is reported as agreement or correspondence between a shortened and the full measure, not as validity against an independent criterion. This correspondence increased monotonically with k, exceeding a point estimate of r = 0.90 for the first time at k = 7, although the 95% confidence interval at k = 7 (0.76 to 0.99) still included values well below 0.90 (k = 4: r = 0.72; k = 5: r = 0.77; k = 6: r = 0.85; k = 7: r = 0.94; k = 8: r = 0.99; Supplementary Table S4; Figure 5).

For the k = 7 index specifically, agreement with the full-protocol index was further characterised with the intraclass correlation coefficient and the Bland-Altman method: ICC = 0.96, mean bias = −0.67 percentage points (the seven-shuttle index running slightly lower than the full index on average), and 95% limits of agreement of −2.58 to 1.25 percentage points. This pattern, a high ICC with a small but non-zero systematic bias and limits of agreement spanning roughly four percentage points on a fatigue index whose sample mean was 7.94%, indicates close but not exact reproduction of the full-protocol ranking, consistent with a nested measure rather than an independently validated one. The k = 7 threshold also closely matched the quadratic-model maximum identified independently at shuttle 7.27 (95% CI 6.15–11.34) and marked on the same trajectory in Figure 2, two analyses constructed from different aspects of the data that converge on a similar shuttle count.

### 3.7. Exploratory Association with Jump-Derived and Physiological Indices

At the full ten-shuttle protocol length, no jump or physiological variable survived correction for multiple comparisons (minimum corrected q = 0.33). An exploratory, post hoc search across k = 2 to 10 identified three variables with correlations surviving correction within their own nine-test family (Supplementary Table S5).

These were bilateral CMJ height (k = 4; ρ = 0.88, q = 0.007), squat jump height (k = 4; ρ = 0.86, q = 0.014), and dominant-leg unilateral CMJ height (k = 2; ρ = 0.77, q = 0.033), all positive and consistent in direction and magnitude with the correlations reported for the identically structured 4/2/4/2/4 m fencing-specific change-of-direction test [27]. The associations for bilateral CMJ height and squat jump height at k = 4 are illustrated in Figure 6.

Because k was selected after inspecting the data for each variable independently, and correction was applied only within each variable’s own search rather than across the full set of variables and k values jointly, these associations are reported as hypothesis-generating rather than confirmatory.

## 4. Discussion

Five results anchor this study, presented here in order of evidential strength. First, and most robustly, movement speed deteriorated systematically across the Day 3 shuttle protocol while touch score showed no detectable group-level decline, an internally consistent speed-accuracy dissociation supported by a large paired effect for shuttle time (dz = 1.09). This decline documents performance fatigability; because no independent assessment of neuromuscular function and no control condition were included, it does not identify the physiological or neuromuscular mechanisms responsible for the slower shuttles [8]. Second, on the staged Day 2 task, no detectable difference in touch accuracy was observed across the approximate exercise-intensity states, including the condition initiated at approximately 85% HRmax, in contrast to the pattern reported for basketball shooting under a comparable staged design [18,19] and to the decline in tennis groundstroke accuracy after high-intensity fatigue [17]; gesture complexity, not heart-rate condition, was associated with accuracy, with the lunge less accurate than the straight thrust throughout, and no evidence emerged that the two gestures responded differently to heart-rate condition. Third, a seven-shuttle version of the protocol closely reproduced the ranking generated by the full ten-shuttle fatigue index (ICC = 0.96), identified by two analyses constructed from different aspects of the data; this is agreement between a nested and a full measure, which identifies seven shuttles as a candidate protocol length rather than a validated replacement for the full test. Fourth, and among the more original observations here, a four-shuttle index and the seven-shuttle index may capture different aspects of the response: the shorter index related, in exploratory analyses, to lower-limb power, while the longer index tracked cumulative, repeated-effort fatigue, suggesting these are complementary rather than competing descriptions of the same phenomenon. Fifth, convergent-validity evidence linking Day 3 target performance to an independent Day 2 assessment of the same skill was positive in direction but not statistically conclusive at this sample size.

Two qualifications apply to the accuracy findings. First, the absence of a statistically significant decline in touch score does not demonstrate that accuracy was preserved: with ten athletes and marked shuttle-to-shuttle variability (Figure 3), only a large decrement would have been detectable, and showing that accuracy remained within a practically acceptable range would require an equivalence test against a margin defined a priori [51]. The results are therefore described as no detectable decline in touch accuracy. Second, this pattern is consistent with the fencing literature. Fencers facing increasing task difficulty on an instrumented simulator became slower while largely preserving touch accuracy [9], precision loss under fatigue was observed in sub-elite but not in Olympic-level fencers [12], and a decline in accuracy was reported in city-level épée fencers [16]; the heterogeneous individual trajectories in Figure 3 suggest that such level-dependent responses may coexist within a mixed-level cohort such as the present one. Marked between-athlete differences have likewise been reported in the response of choice reaction time to a 12-week program in U20 foil fencers, together with only modest changes in heart rate [15], which supports reporting individual responses alongside group means in small fencing samples. Finally, the mean lunge velocity observed on Day 2 (3.44 ± 1.11 m·s^−1^; Table 3) was of the same order as that reported for adolescent fencers of a national academy (3.35 m·s^−1^), in whom lower-limb power, expressed by standing broad jump, was the strongest predictor of both lunge velocity and change-of-direction speed [52]; this is coherent with the exploratory association between jump height and the early-shuttle fatigue index observed here.

The absence of a detected heart-rate effect on Day 3 accuracy deserves closer reasoning rather than being read as a separate, unexplained null result. Expressed relative to each athlete’s own maximal heart rate, the continuous Day 3 protocol already held athletes at a mean intensity (84.60 ± 3.56% of HRmax) closely matching the Day 2 85%HRmax condition, and at a peak intensity (93.45 ± 6.00% of HRmax) that exceeded it, with nine of ten athletes reaching at least 85%HRmax at some point during the test (Figure 4). Because Day 2 found no detectable accuracy decrement across the tested exercise-intensity states, including the condition initiated at approximately 85% HRmax, the Day 3 protocol offers little remaining dynamic range in which a heart-rate threshold effect could appear: athletes spend most of the test already inside, or above, the zone Day 2 examined. Read this way, the Day 3 finding is not an independent null result but a direct, coherent extension of the Day 2 result, and it reframes the open question: whether a fencing-specific accuracy threshold exists at all can only be tested by sampling heart-rate zones above those reached here, which neither Day 2 nor Day 3 did. A staged-intensity design extending beyond 85%HRmax, rather than a further continuous shuttle protocol, is therefore the more informative next step for this specific question.

This reasoning depends on individually normalised %HRmax, and that normalisation carries its own uncertainty that should be stated plainly rather than absorbed into the headline percentages. Expressing Day 3 heart rate relative to each athlete’s own Day 1 maximum assumes that the Day 1 value accurately captured each athlete’s true physiological ceiling, an assumption a single, field-based Beep Test cannot guarantee: motivation, pacing, and test-termination criteria all affect whether a genuine maximum is reached, and two of the ten athletes recorded a Day 3 peak heart rate that exceeded their own Day 1 value, direct evidence within this sample that the Day 1 ceiling was not always captured. Because this kind of measurement error inflates %HRmax specifically for the athletes whose true maximum was underestimated on Day 1, the individual positions shown in Figure 4, and to some extent the precise sample percentages (84.60%, 93.45%), should be read as approximate rather than exact, and part of the apparent between-athlete spread may reflect inconsistency in how completely Day 1 captured each athlete’s ceiling rather than genuine between-athlete variability in relative effort. This concern is partly, but not fully, addressed by the fact that the raw, non-normalised heart-rate analysis (mean heart rate in beats·min^−1^, reported above) produced a materially identical pattern to the %HRmax analysis; a marked discrepancy between the two would have suggested the normalisation itself was driving the result, and its absence is reassuring without being conclusive. A laboratory-based, gas-exchange-verified determination of maximal heart rate would remove this specific source of uncertainty in future work.

Regarding validation specifically, three lines of evidence were examined, and they differ in what they can and cannot show. Agreement between the shortened and full ten-shuttle fatigue index was the strongest and clearest finding (ICC = 0.96), but because the shortened index is mathematically nested within the full index, this demonstrates reproducibility of a nested measure rather than validity against an independent criterion; we did not attempt to validate the absolute fatigue index as a reliable measure in its own right, a claim its known statistical instability [21,22] would not support, but examined instead whether a shortened version reproduces the athlete ranking generated by the conventional full-protocol calculation, a narrower and better-supported claim. Exploratory associations with lower-limb power were identified for a four-shuttle index after a post hoc search across k and should be treated as hypothesis-generating pending confirmatory testing in an independent sample. Convergent validity between the two independent measurements of target performance, Day 2 under controlled conditions and Day 3 under continuous repeated-effort fatigue, was positive in direction for accuracy, precision, and PEI alike, and strengthened when a single extreme outlier was excluded, but did not reach significance with ten athletes. This pattern is compatible with a moderate positive relationship, but the estimates remain imprecise given the small sample and cannot be interpreted as evidence of convergent validity; the achieved power calculation reported in the Statistical Analysis indicates that a true correlation of this magnitude (ρ ≈ 0.4 to 0.5) would require a considerably larger sample to detect reliably, and the individualised, non-standardised target distance used on Day 2 may have added measurement noise to this specific comparison independently of sample size.

The one correlation that reached nominal significance before correction, Day 3 PEI with dominant-hand grip strength, in the counter-intuitive direction that stronger grip corresponded to worse target performance, did not survive either outlier exclusion or multiplicity correction, and should not be treated as a genuine finding; it is reported for transparency and as a candidate hypothesis, not a conclusion.

What would most directly complete the validation of the shuttle-with-target test is a formal test-retest of the Day 3 protocol, which was not performed in the present study. Repeating the Day 3 procedure under identical conditions in an independent session would allow computation of test-retest intraclass correlation coefficients, standard error of measurement, and Bland-Altman limits of agreement for every Day 3 outcome, time, score, accuracy, precision, PEI, and the fatigue index itself, which is the single most direct and standard form of evidence for a newly proposed field test. Concretely, we recommend three steps to complete the validation package: first, conduct a dedicated test-retest of the Day 3 protocol to establish reliability of every outcome; second, replicate the Day 2-to-Day 3 convergent-validity correlations in a larger, ideally multi-site sample, since the direction and approximate magnitude observed here are encouraging but underpowered; and third, standardize the target execution distance on Day 2, currently adapted individually per athlete, so that Day 2 and Day 3 accuracy measures are unambiguously comparable rather than only approximately so.

On protocol length, the convergence between the Day 3 time-trajectory maximum and the shortened-index agreement threshold is stronger evidence than either analysis alone that a seven-shuttle version reproduces this test’s fatigue signature, although the wide bootstrap confidence interval around the estimated maximum (6.15 to 11.34) means this should be read as the best available estimate from this sample rather than a precisely located physiological landmark. Moreover, because the seven- and ten-shuttle indices share seven of their ten observations, a strong association between them is partly expected by construction, and fatigue indices have shown weak reliability in repeated-sprint testing [21,23]; seven shuttles should therefore be regarded as a candidate protocol length, whose adoption as a practical alternative to the full test requires independent test-retest reliability and further validation. The jump-derived correlations tell a complementary but distinct, and more original, story: CMJ and SJ related most strongly, in exploratory analyses, to the fatigue index computed from the first two to four shuttles, not the full protocol. A four-shuttle index and a seven-shuttle index therefore appear to capture related but different phenomena: the former showing exploratory associations with lower-limb power and the latter more closely reproducing the cumulative fatigue trajectory that the full protocol was built to assess. If replicated, this distinction, rather than a single recommended shuttle count, may be the more useful methodological contribution of this work.

### Limitations

The small sample (*n* = 10) limits the precision of every estimate, as quantified in the Statistical Analysis. Maximal heart rate, and every %HRmax value derived from it, rested on a single field-based Beep Test. The target execution distance on Day 2 was not standardized, which may have added measurement noise to the Day 2-to-Day 3 convergent-validity comparison independently of sample size, and heart rate during the five Day 2 trials themselves, as opposed to at condition onset, was not independently re-verified. The Day 2 intensity states were also reached through different preceding exercise exposures and should be regarded as approximate. No independent assessment of neuromuscular fatigue and no control condition were included, so the mechanisms underlying the slower shuttles cannot be identified, and the fixed 8 s recovery reproduces the work-recovery structure of épée more closely than that of foil [33]. No test-retest of the Day 3 shuttle-with-target protocol was performed, preventing computation of test-retest reliability for its outcomes; this is disclosed as the principal open item for the next stage of this work. PEI is treated throughout as an exploratory composite, since Day 2 cells were built from only five trials each, and the mean score (equivalently, MAE) and SD are score-based proxies of accuracy and precision derived from a discrete, zone-based scale. Finally, the fatigue index itself carries a documented reliability ceiling [20,21,22,23], which is why it was used here as a ranking criterion for protocol-length comparison rather than as a validated absolute measure.

## 5. Conclusions

In this exploratory sample, a continuous repeated-shuttle task produced performance fatigability, expressed as a robust, non-linear increase in movement time, with no detectable group-level decline in touch score, and a directly staged heart-rate task found no detectable accuracy decrement up to 85% of maximal heart rate. A seven-shuttle version of the protocol closely reproduced the ranking of the full ten-shuttle fatigue index (ICC = 0.96), identifying seven shuttles as a candidate protocol length rather than a validated replacement for the full test, and, in exploratory analyses, a four-shuttle index correlated with lower-limb power, suggesting these two shortened indices may capture related but distinct aspects of performance fatigability. Preliminary convergent-validity evidence linking Day 3 target performance to an independent, controlled target task was positive in direction but not statistically conclusive at this sample size. These findings should be read as a preliminary, hypothesis-generating characterization of the shuttle-with-target test rather than a completed validation; a dedicated test-retest of the Day 3 protocol, together with replication of the convergent-validity findings in a larger sample, is the most direct next step before the test is adopted for applied decision-making.

## Figures and Tables

**Figure 1:**
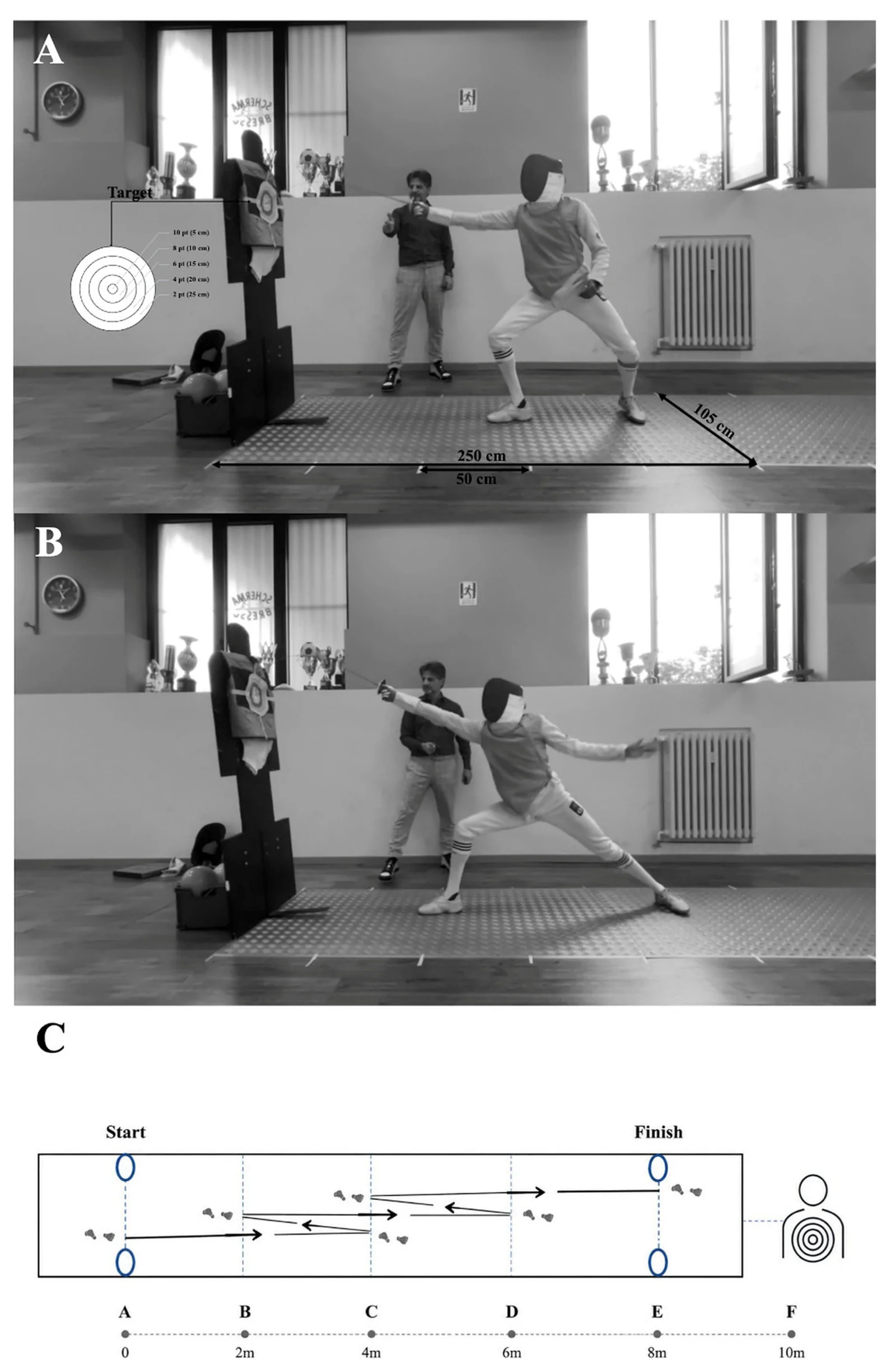
Day 2 target task and Day 3 shuttle-with-target protocol. (**A**) Straight thrust (tiro dritto/braccio) from the en-garde position, target positioned 250 cm from the starting line, with the five-zone graded target shown at left (10 to 2 points, 5 to 25 cm diameter). (**B**) Lunge (affondo); foot-placement references at 50 cm and 105 cm are marked on the strip. (**C**) Schematic of the 16 m, 4–2–4–2–4 m shuttle course used on Day 3 (points A to F at 0, 2, 4, 6, 8, and 10 m), ending in a lunge to the graded target.

**Figure 2:**
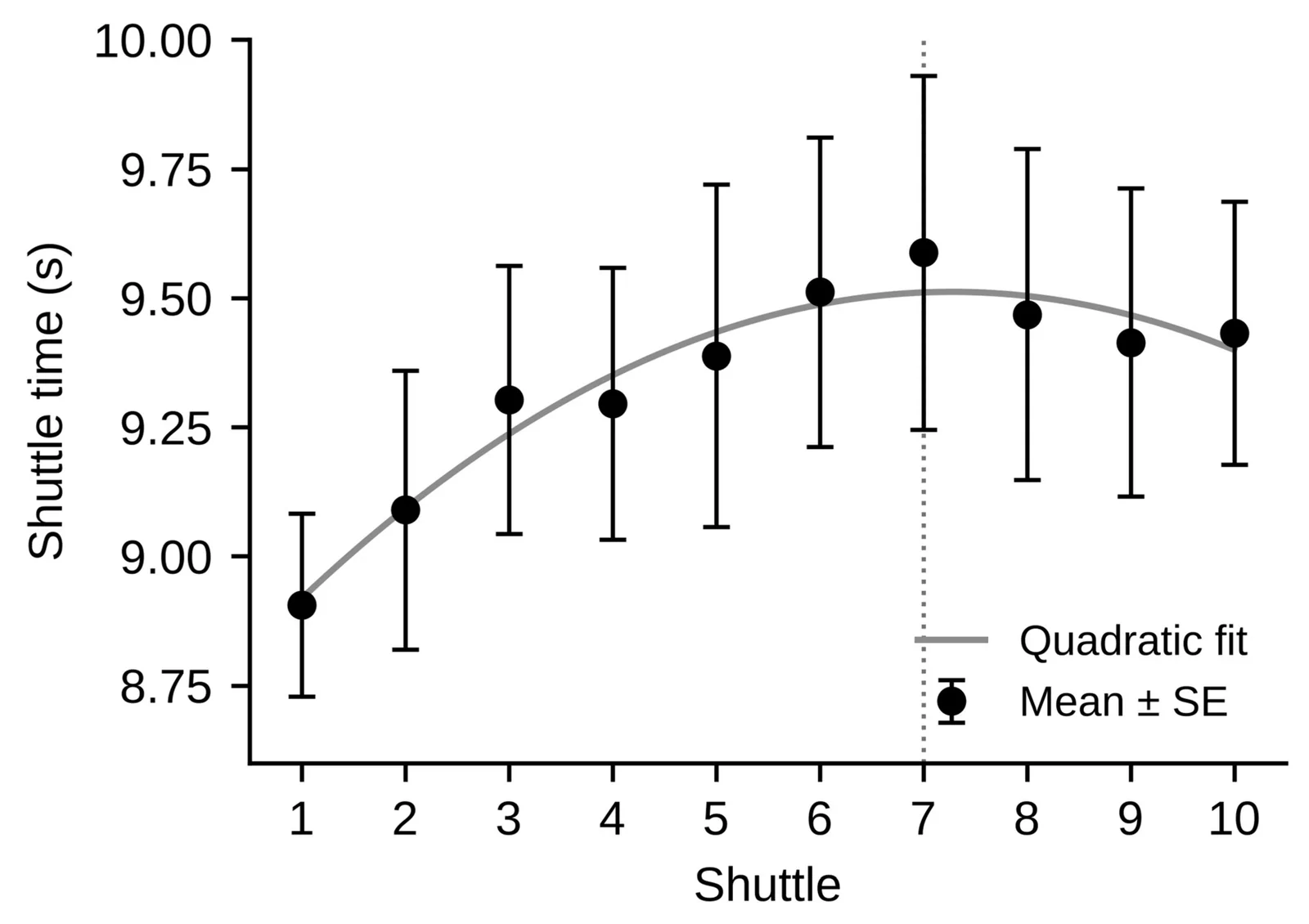
Mean shuttle time (± SE) across the Day 3 ten-shuttle protocol, with the fitted quadratic trajectory from the linear mixed-effects model and the k = 7 shortened-index agreement threshold identified in the shuttle-number simulation. The curve reaches its estimated maximum, not a physiological plateau, at shuttle 7.27. Linear term: β = 0.054 s per shuttle, *p* < 0.001; quadratic term: β = −0.015, *p* = 0.005; likelihood-ratio test, quadratic vs. linear model: χ^2^(1) = 7.58, *p* = 0.006. The dotted vertical line marks k = 7.

**Figure 3:**
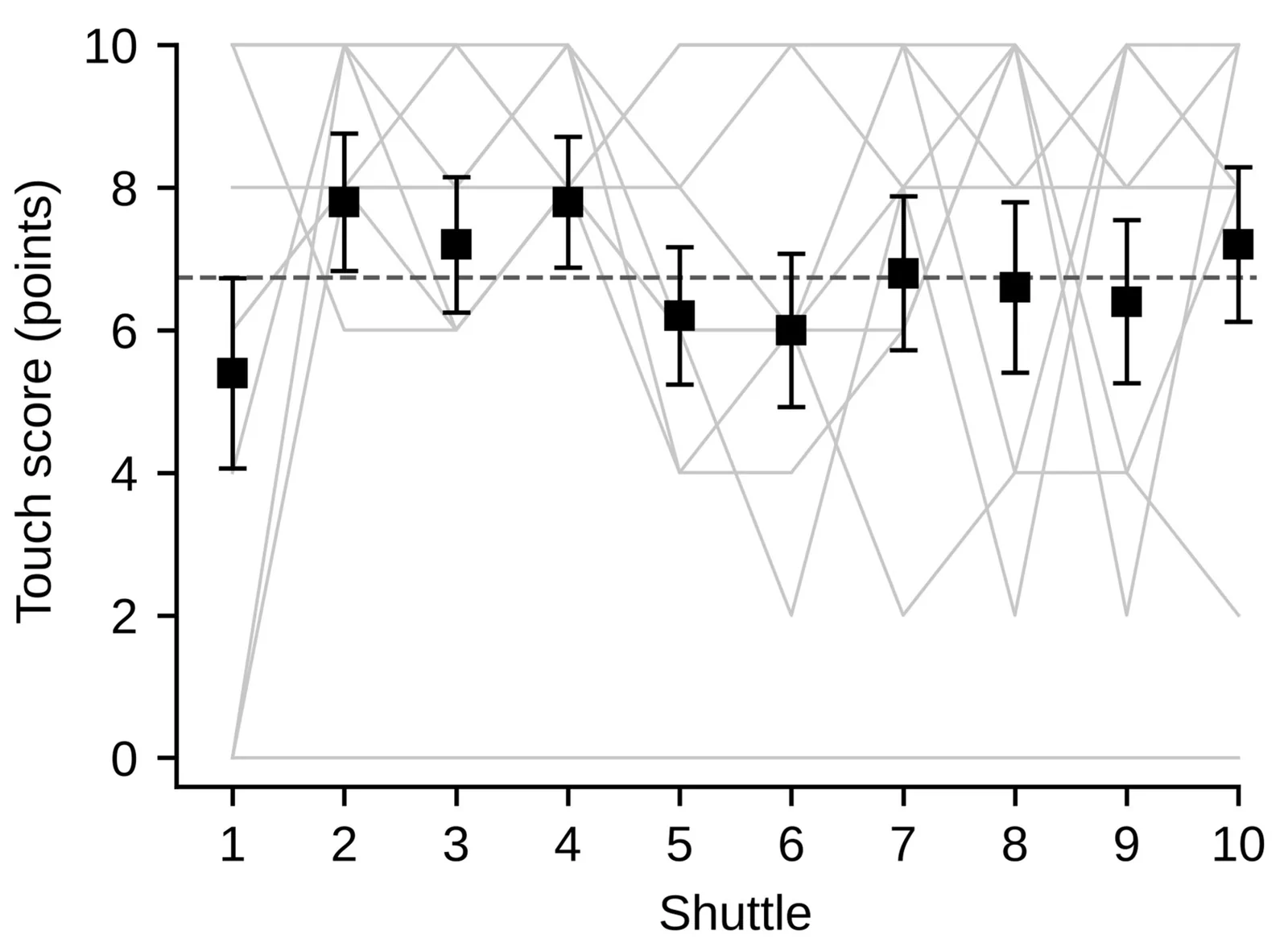
Touch score across the Day 3 ten-shuttle protocol: group mean (± SE, black squares) and individual athlete trajectories (thin grey lines, *n* = 10, anonymised). The dashed line marks the overall group mean. Linear mixed model for the group-level trend: β = 0.001, *p* = 0.988.

**Figure 4:**
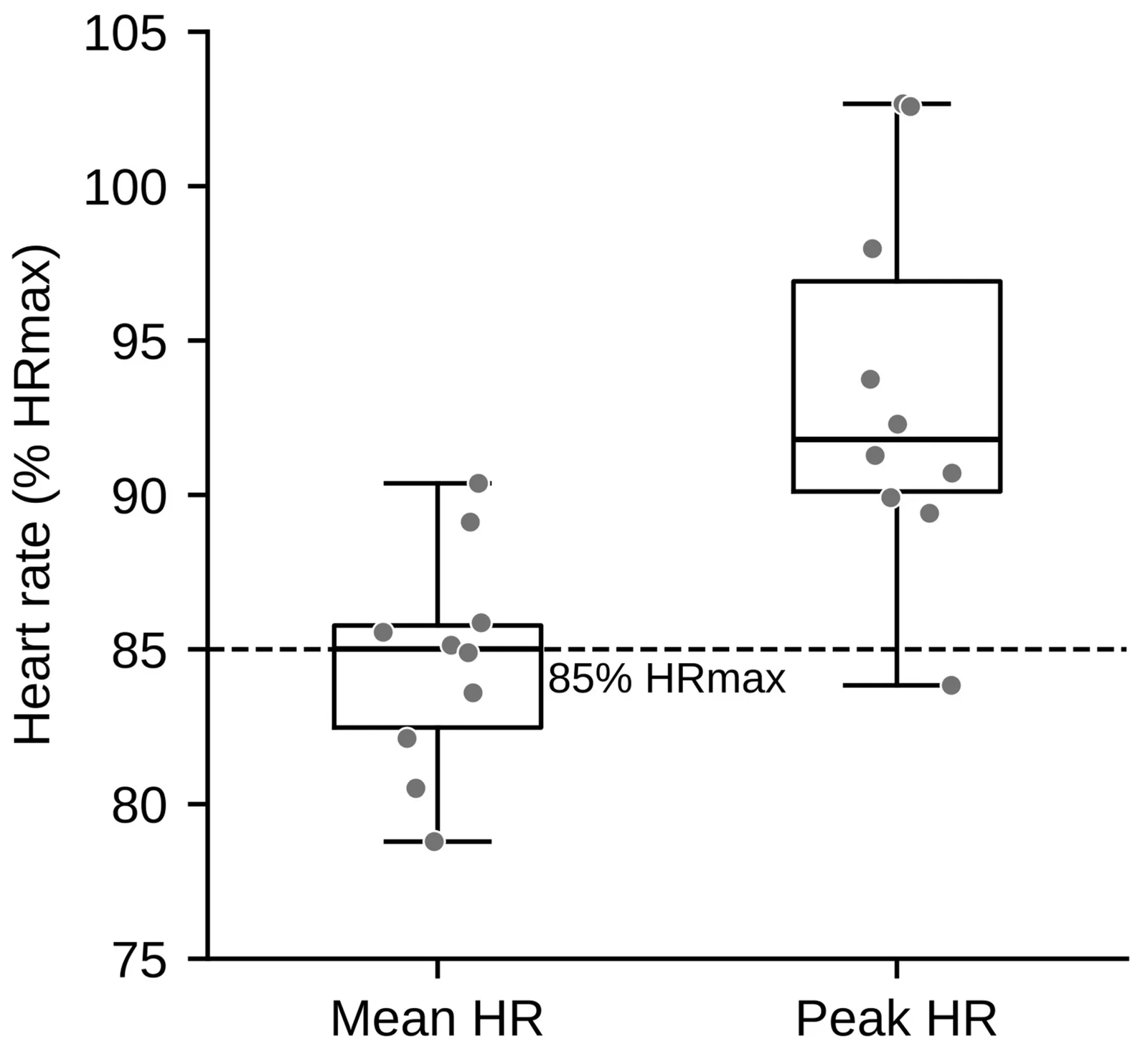
Distribution of Day 3 mean and peak heart rate, expressed as a percentage of each athlete’s own Day 1 maximal heart rate, relative to the Day 2 85%HRmax condition (dashed horizontal line). Values above 100% reflect athletes whose Day 3 peak exceeded their Day 1 recorded maximum, most plausibly because the Day 1 field test underestimated their true physiological ceiling (see Discussion), and should not be read as heart rate literally exceeding a true maximum. Boxes show the median and interquartile range; whiskers show the full range; individual athlete values are shown as anonymised grey points (*n* = 10, jittered horizontally for visibility, no athlete identifiers attached). Mean ± SD: mean heart rate 84.60 ± 3.56% and peak heart rate 93.45 ± 6.00% of maximal heart rate; nine of ten athletes reached at least 85% HRmax, and two exceeded their Day 1 recorded maximum.

**Figure 5:**
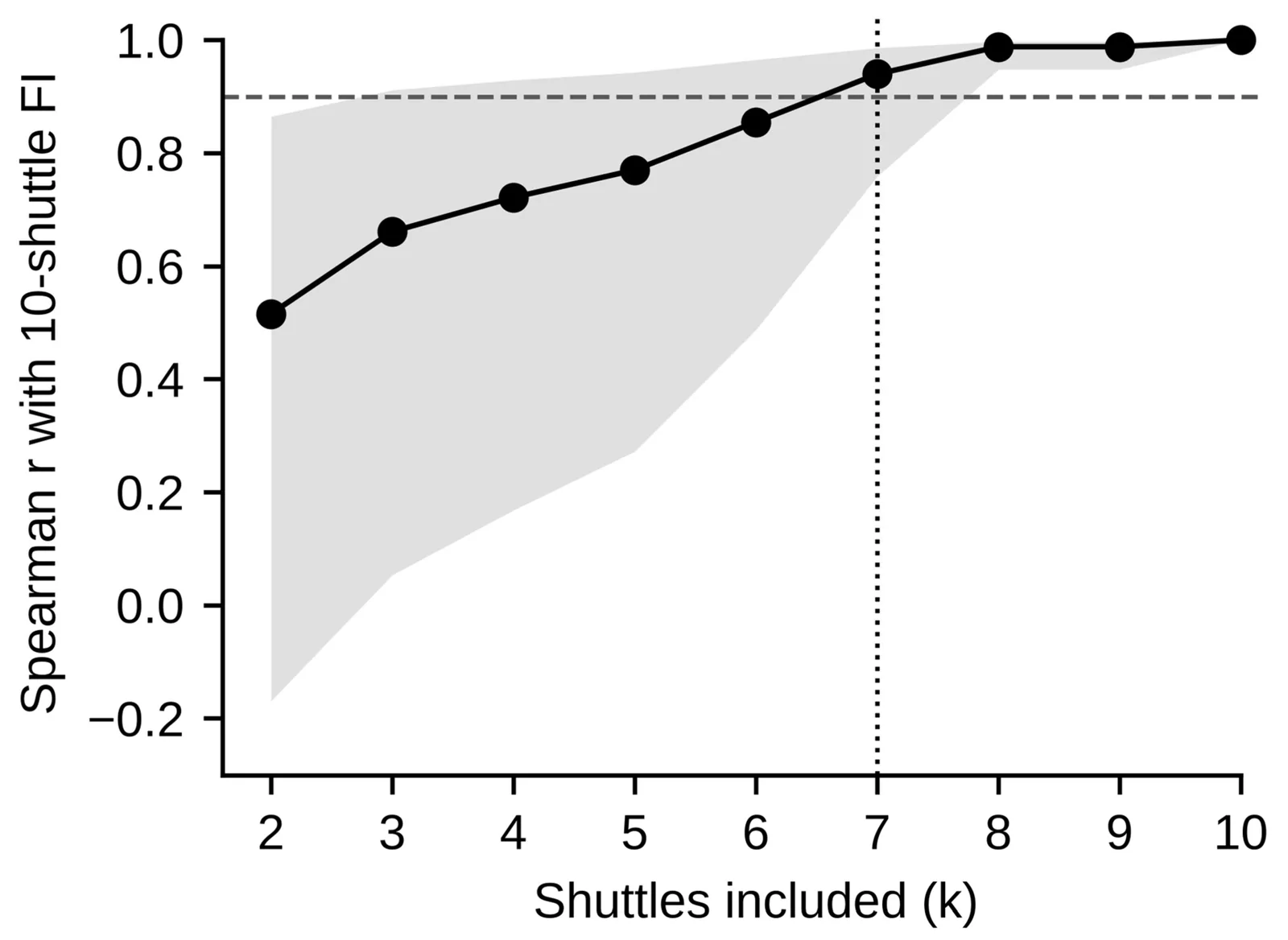
Agreement of the fatigue index computed from the first k Day 3 shuttles with the full ten-shuttle fatigue index, as a function of k. Because the shortened index is nested within the full index, this reflects reproducibility rather than independent validity. Point estimates (Spearman r) are shown with 95% confidence intervals (Fisher z; shaded band); *p*-values are reported in Supplementary Table S4. The dashed line marks r = 0.90 and the dotted line marks k = 7, the first k at which the point estimate exceeds it.

**Figure 6:**
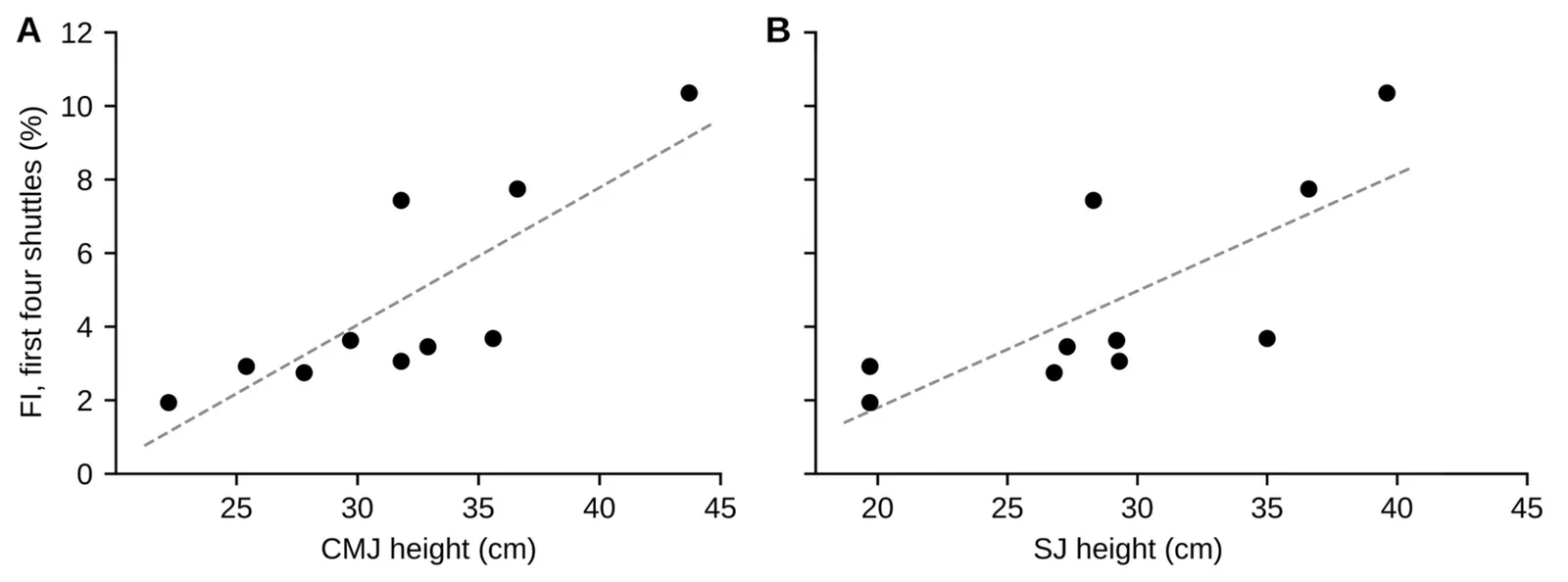
Exploratory, post hoc association between the Day 3 fatigue index computed from the first four shuttles and countermovement jump height (**A**) and squat jump height (**B**). (**A**) Spearman ρ = 0.88, *p* = 0.001, q = 0.007; (**B**) ρ = 0.86, *p* = 0.002, q = 0.014 (q, Benjamini-Hochberg-corrected within each variable family); the dashed line is a linear trend shown for visualisation only, not the basis for inference.

**Table 1:** Anthropometric, physical capacity, and neuromuscular characteristics of the sample (*n* = 10).

Variable	Mean ± SD	*n*
Age (years)	21.20 ± 4.90	10
Height (cm)	174.70 ± 9.25	10
Body mass (kg)	69.57 ± 12.18	10
Leg length (cm)	83.80 ± 5.31	10
Dominant-hand grip strength (kg)	43.95 ± 8.19	10
Non-dominant-hand grip strength (kg)	40.10 ± 6.78	10
Maximal heart rate (beats·min^−1^)	195.90 ± 8.52	10
Estimated V̇O_2_max (mL·kg^−1^·min^−1^)	42.33 ± 9.00	9
Squat jump height (cm)	29.15 ± 6.56	10
Countermovement jump height (cm)	31.75 ± 6.09	10
Dominant-leg unilateral CMJ height (cm)	16.47 ± 4.18	10
Non-dominant-leg unilateral CMJ height (cm)	13.66 ± 4.60	10
Between-limb asymmetry index	0.11 ± 0.09	9
CMJ/SJ ratio	1.10 ± 0.09	10
Elasticity Index (%)	8.59 ± 7.24	10
Musculotendinous stiffness (%)	9.56 ± 6.98	9

CMJ, countermovement jump; SJ, squat jump. Maximal heart rate and V̇O_2_max are field-based estimates from the 20 m Shuttle Run Test (Beep Test), not laboratory-, gas-exchange-verified values (see Discussion). One athlete’s recorded V̇O_2_max value of exactly 0 was treated as not measured (*n* = 9); the asymmetry index and musculotendinous stiffness likewise excluded one athlete each with an implausible recorded value of exactly 0 (*n* = 9). The CMJ/SJ ratio is the dimensionless ratio recomputed from the recorded CMJ and SJ heights.

**Table 2:** Mean touch score, accuracy (MAE), and within-athlete precision (SD) on Day 2, by heart-rate condition and gesture type.

Heart-Rate Condition	Gesture	Mean Score	SD Score	MAE	*n* Trials
Baseline	Straight thrust	8.86	1.22	1.14	49
Baseline	Lunge	8.04	1.71	1.96	46
50%HRmax	Straight thrust	8.44	1.54	1.56	54
50%HRmax	Lunge	7.93	1.69	2.07	54
85%HRmax	Straight thrust	8.63	1.52	1.37	51
85%HRmax	Lunge	8.19	1.27	1.81	52

MAE, mean absolute error from the maximum score (10), trial-level; because no score can exceed 10, MAE equals 10 minus the mean score and does not provide information independent of it. SD is within-athlete precision (variability across trials within each cell), not between-athlete variability. Values pooled across the ten athletes; realized trial counts (*n* = 306 total) differed slightly from the nominal protocol (300) as detailed in Methods.

**Table 3:** Video-derived kinematic variables, by gesture type.

Variable	Straight Thrust (Mean ± SD)	Lunge (Mean ± SD)
Reaction time (s)	0.04 ± 0.04	0.04 ± 0.03
Autonomous execution time (s)	0.32 ± 0.14	0.47 ± 0.17
Upper–lower limb temporal difference (s)	0.00 ± 0.00	0.12 ± 0.13
Execution distance (m)	1.33 ± 0.10	1.70 ± 0.21
Mean autonomous velocity (m·s^−1^)	4.40 ± 1.41	3.44 ± 1.11

Reaction time, interval from the examiner’s verbal signal to first observable wrist movement. Autonomous execution time, interval from first arm movement to weapon–target contact, excluding reaction time. The upper–lower limb temporal difference is, by definition, uniformly 0 for the straight thrust, which involves no lower-limb displacement. Values pooled across all trials and all ten athletes (*n* = 154 straight-thrust trials, *n* = 152 lunge trials).

## Data Availability

The data supporting the findings of this study are available from the corresponding author upon reasonable request.

## Supplementary Materials

The following supporting information can be downloaded at: https://www.mdpi.com/article/10.3390/sports14100459/s1. Table S1: Descriptive statistics per shuttle on Day 3, pooled across the ten athletes (*n* = 10 per shuttle); Table S2: Session-level summary: fatigue index, heart rate, and rating of perceived exertion (*n* = 10); Table S3: Convergent-validity correlations between Day 3 shuttle-with-target performance and Day 2 target performance, and between Day 3 Performance Error Index (PEI) and Day 1 physical capacity measures; Table S4: Agreement between the fatigue index computed from the first k Day 3 shuttles and the fatigue index computed from the full ten-shuttle protocol; Table S5: Strongest exploratory, post hoc association between the Day 3 fatigue index and each jump or physiological variable across the nine values of k tested (k = 2 to 10).
