# Supplementary material for: Speed-Accuracy Dissociation in Fencing: Performance Fatigability Without a Detectable Decline in Touch Accuracy: An Exploratory Study
Source: Sports (Basel). 2026 Oct 15;14(10):459. doi: 10.3390/sports14100459 (PMC13709925; doi:10.3390/sports14100459)
Supplement: Supplementary file 1 [file sports-14-00459-s001.zip › sports-4583783-supplementary.pdf]

Supplementary Materials

Speed-Accuracy Dissociation in Fencing: Performance Fatigability Without a Detectable Decline in Touch Accuracy. An Exploratory Study

Mirko Colombo, Gian Mario Migliaccio, Domenico Martone and Johnny Padulo

Table S1. Descriptive statistics per shuttle on Day 3, pooled across the ten athletes (n = 10 per shuttle).

Shuttle	Mean time (s)	SD time (s)	Mean score (points)	SD score (points)	MAE (points)	Accuracy (%)
1	8.91	0.56	5.40	4.22	4.60	54.00
2	9.09	0.85	7.80	3.05	2.20	78.00
3	9.30	0.82	7.20	3.01	2.80	72.00
4	9.30	0.83	7.80	2.90	2.20	78.00
5	9.39	1.05	6.20	3.05	3.80	62.00
6	9.51	0.95	6.00	3.40	4.00	60.00
7	9.59	1.08	6.80	3.43	3.20	68.00
8	9.47	1.01	6.60	3.78	3.40	66.00
9	9.41	0.94	6.40	3.63	3.60	64.00
10	9.43	0.80	7.20	3.43	2.80	72.00

MAE, mean absolute error from the maximum score (10). Because no score can exceed 10, MAE equals 10 minus the mean score, and Accuracy (%) = (1 − MAE / 10) × 100 equals ten times the mean score; neither provides information independent of the mean score, and both are shown only for comparison with studies that report them. SD columns are between-athlete dispersion at each shuttle (n = 10 athletes), a distinct quantity from the within-athlete precision defined in Methods and used for PEI.

Table S2. Session-level summary: fatigue index, heart rate, and rating of perceived exertion (n = 10).

Variable	Mean ± SD
Fatigue index, full protocol (%)	7.94 ± 4.13
Mean heart rate (beats·min^{-1})	165.60 ± 7.32
Peak heart rate (beats·min^{-1})	182.90 ± 11.42
RPE, pre-session (Borg CR10, points)	0.00 ± 0.00
RPE, 30 min post-session (Borg CR10, points)	6.10 ± 0.99

Fatigue index = 100 × (ΣT_TOT / [best time × 10]) − 100, used as a ranking criterion for protocol-length comparison rather than a validated absolute measure (see Discussion). Pre-session RPE was 0 for all ten athletes, a valid value on the Borg CR10 scale (“nothing at all”) at a resting baseline, not a missing value.

Table S3. Convergent-validity correlations between Day 3 shuttle-with-target performance and Day 2 target performance, and between Day 3 Performance Error Index (PEI) and Day 1 physical capacity measures.

Comparison	ϱ (n = 10)	P	ϱ (outlier excluded, n = 9)	P	Corrected q
Day 3 vs. Day 2 accuracy (MAE)	0.47	0.167	0.55	0.129	0.500
Day 3 vs. Day 2 precision (SD)	0.30	0.393	0.43	0.252	0.556
Day 3 vs. Day 2 PEI	0.39	0.267	0.44	0.232	0.533
Day 3 PEI vs. dominant-hand grip strength	0.68	0.030	0.61	0.079	0.177
Day 3 PEI vs. non-dominant-hand grip strength	−0.11	0.763	–	–	0.763
Day 3 PEI vs. estimated	−0.28	0.463	–	–	0.556

$\dot{V}O_2max$ (n = 9)

PEI, Performance Error Index = $\sqrt{(MAE^2 + SD^2)} = \sqrt{((10 - \text{mean score})^2 + SD^2)}$, an exploratory composite (Day 2 cells built from five trials each). Because MAE equals 10 minus the mean score on both days, the accuracy correlation is identical when computed on mean score. Day 2 values pooled across all three heart-rate conditions and both gesture types. "Outlier excluded" removes the single athlete with a constant zero score across all ten Day 3 shuttles. q, Benjamini-Hochberg-corrected P-value across the six-test convergent-validity family (full-sample correlations); no correlation survived correction (minimum q = 0.177).

Table S4. Agreement between the fatigue index computed from the first k Day 3 shuttles and the fatigue index computed from the full ten-shuttle protocol.

Shuttles used (k)	r	95% CI	P
2	0.52	−0.17, 0.86	0.128
3	0.66	0.05, 0.91	0.038
4	0.72	0.17, 0.93	0.019
5	0.77	0.27, 0.94	0.009
6	0.85	0.49, 0.97	0.002
7	0.94	0.76, 0.99	< 0.001
8	0.99	0.95, 1.00	< 0.001
9	0.99	0.95, 1.00	< 0.001
10	1.00	–	–

FI, fatigue index = 100 × (ΣT_TOT / [best time × k]) − 100. r = Spearman correlation between the fatigue index computed from the first k shuttles and that computed from all ten shuttles (n = 10 athletes); because the shortened index is nested within the full index, r reflects agreement, not independent criterion validity, and is 1.00 by definition at k = 10. 95% CI by Fisher z transformation. For the k = 7 index: ICC (two-way random, absolute agreement) = 0.96; Bland-Altman mean bias = −0.67 percentage points; 95% limits of agreement = −2.58 to 1.25 percentage points.

Table S5. Strongest exploratory, post hoc association between the Day 3 fatigue index and each jump or physiological variable across the nine values of k tested (k = 2 to 10).

Variable	Best k	ρ	P	Corrected q (within variable)
CMJ (cm)	4	0.88	0.001	0.007
SJ (cm)	4	0.86	0.002	0.014
CMJ DX (cm)	2	0.77	0.009	0.033
Musculotendinous stiffness (%)	2	−0.55	0.098	0.363
Estimated $\dot{V}O_2max$ (mL·kg^{-1}·min^{-1})	5	0.76	0.017	0.096

CMJ, countermovement jump; SJ, squat jump; CMJ DX, dominant-leg unilateral countermovement jump. k was selected post hoc for each variable after inspecting all nine values; q is the Benjamini-Hochberg-corrected P-value within each variable's own nine-test family only, not across all variables and k values jointly. Only the first three rows survive correction at q < 0.05; all are reported as hypothesis-generating.
